# *Glandless*, a tomato HD‐ZIP transcription factor, is important for the gland formation of type VI trichomes

**DOI:** 10.1111/tpj.70308

**Published:** 2025-07-02

**Authors:** Pietro Zocca, Eva van Doore, Alwin J.M. Roovers, Joris J. Glas, Maarten Uittenbogaard, Maarten G. Verlaan, Zeger van Herwijnen, Michel A. Haring, Robert C. Schuurink

**Affiliations:** ^1^ Green Life Sciences Research Cluster, Swammerdam Institute for Life Sciences University of Amsterdam Amsterdam 1098 XH The Netherlands; ^2^ Rijk Zwaan Breeding B.V. Burgemeester Crezéelaan 40 2678 ZG De Lier The Netherlands

**Keywords:** glandular trichome, tomato, terpenes, transcription factor, acylsugars

## Abstract

Tomato (*Solanum lycopersicum*) is a model plant to study glandular trichome development and their specialized metabolism, and several transcription factors (TF) regulating these intertwined traits and their network have been functionally characterized. Among them are members of the homeodomain leucine zipper subfamily IV (HD‐ZIP IV). Here, we study a tomato EMS‐mutant line, *glandless*, presenting mutant, glandless type VI trichomes with a consequential reduction in volatile terpene levels. This mutant trichome also has some morphological characteristics of a type IV trichome. The *glandless* mutant has altered trichome densities, and acylsugar biosynthesis is slightly increased. As verified via virus‐induced gene silencing (VIGS), the gene underlying this phenotype is SlHDZ38, the first member of HD‐ZIP subfamily I found to regulate the development and specialized metabolism of glandular trichomes. Additionally, we show that the expression of an intricate network of known trichome‐related regulatory TFs and biosynthetic enzymes is affected by the *glandless* mutation. Overall, our results contribute to the elucidation of the network of TFs controlling tomato trichomes.

## Significance Statement

We studied a tomato EMS‐mutant plant, *glandless*, that has glandless type VI glandular trichome. The gene underlying this phenotype is a member of the HD‐ZIP subfamily I, which regulates the development and specialized metabolism of type VI glandular trichomes.

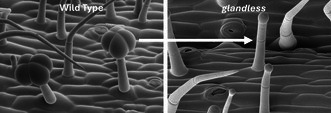

How the formation of glands of glandular trichomes is regulated or controlled is not well understood. By selecting a tomato mutant with type VI trichomes without a gland and identifying the responsible *Glandless* gene, we now know a key player in this process.

## INTRODUCTION

Among adaptations in vascular plants to interact with the environment, the hair‐like structures called trichomes are found in approximately one‐third of the species with vastly diversified morphologies (Fahn, 2000; Payne, 1978). Glandular trichomes and their metabolites are critical for plants to cope with abiotic and biotic stresses and to attract pollinators (Bickford, 2016; Tian et al., 2012; Wagner et al., 2004). Trichome metabolites have also been exploited as high‐value ingredients in the food, cosmetic, and pharmaceutical industries, and as natural pesticides in agriculture (Schilmiller et al., 2008). Tomato (*Solanum lycopersicum*) has become a model species to unravel the regulation of development and specialized metabolism of multicellular glandular trichomes in horticultural crops (Feng et al., 2021). In fact, cultivated tomato accessions and their wild relatives display different combinations and densities of eight types of trichomes, four nonglandular (type II, III, V and VIII) and four glandular (I, IV, VI, VII) (Glas et al., 2012; Luckwill, 1943). The latter are distinguished by their morphology, the number of cells, and the chemical classes of compounds found in them (McDowell et al., 2011). Type VI trichomes are composed of a basal cell, a long stalk cell, a small intermediate cell, and four glandular cells where mostly mono‐ and sesquiterpenes, but also flavonoids and methylketones, are produced (Akhtar et al., 2013; Bleeker et al., 2011; Fridman et al., 2005; Nakashima et al., 2016; Schilmiller et al., 2009). These metabolites are deposited in the intercellular cavity in the middle of them (Bergau et al., 2015; Tissier et al., 2017a).

Recent research has started to unravel the complexity of glandular trichome development in tomato, but there is still much to be learned. Up to now, several tomato transcription factors (TF) have been characterized as regulators of type VI trichome development and metabolism. These mostly belong to the MYB (MYELOBLASTOSIS), bHLH (basic helix loop helix), ZFP (Zinc finger protein), GRAS (GAI, RGA, SCR) and HD‐ZIP (homeodomain leucine zipper) transcription factor (TF) families. The latter, only present in plants, is characterized by a homeobox domain (HD) and a leucine zipper motif (ZIP) and comprises four classes (I–IV) that differ in functional domains (Gao et al., 2015; Zhang et al., 2014). To the subfamily IV belongs SlWO (WOOLLY), a master regulator of trichome type I, III, V, VI, and VII differentiation (Vendemiatti et al., 2017; Wu et al., 2023; Yang, Li, Zhang, Luo, et al., 2011; Yang, Li, Zhang, Wang, et al., 2011). It has been recently suggested that trichome fate during development is controlled by SlWO via a dose‐dependent mechanism. The SlWO protein concentration is regulated by self‐activation of its gene and via negative feedback regulation by SlMTR1 and SlMTR2 (MULTICELLULAR TRICHOME REPRESSOR), previously characterized as trichome regulators with the names SlCYCB2 and SlCYCB3 (Gao et al., 2017; Wu et al., 2023). Low levels of SlWO activate *SlLFS* (*LEAFLESS*), which promotes type VI and VII development, while high SlWO levels activate *SlMX1* (*MIXTA*) and *SlWOX3b* (*WUSCHEL‐RELATED HOMEOBOX*). SlMX1 and SlWOX3b form a complex that promotes type I, III, and V development also by repressing *SlLFS* (Wu et al., 2023). SlWO interacts independently with several of the other characterized trichome regulators. An additional role for SlMTR1 in regulating SlWO at the post‐transcriptional level has been suggested because of their protein–protein interaction (Wu et al., 2023; Yang, Li, Zhang, Luo, et al., 2011; Yang, Li, Zhang, Wang, et al., 2011). Specifically related to type VI glandular trichome development, it has been proposed that under the regulation of jasmonic acid (JA) via SlJAZ2 (JASMONATE ZIM‐DOMAIN) and SlJAZ4, SlWO interacts with SlMYC1 (MYELOCYTOMATOSIS). The resulting SlWO‐SlMYC1 complex binds the promoters of *TPS* (TERPENE SYNTHASE) genes and activates their expression in mature type VI trichome gland cells (Hua et al., 2020; Hua et al., 2021). Additionally, SlWO binds to two C_2_H_2_ zinc finger TFs known to regulate nonglandular trichomes, SlH (HAIR) and SlSH (SPARSE HAIR) (Chang et al., 2018; Chun et al., 2021; Hua et al., 2022; Li et al., 2021; Zheng et al., 2022). It has been suggested that in this three‐way interaction, they cooperate in regulating the formation of multiple types of trichomes (Chang et al., 2018; Chun et al., 2021; Zheng et al., 2022). SlLN (LANATA) is another recently characterized HD‐ZIP IV transcription factor, whose natural mutant shows a higher density of hairy trichomes (type I, III, and V) and lower density of type VI and VII glandular trichomes (Xie et al., 2022). SlLN interacts with SlH and binds to an L1‐box in the SlH promoter, positively regulating its expression. Moreover, similar to SlWO, SlLN binds to the promoter of both *SlMTR1* and *SlMTR2*, activating their expression. SlLN interacts with SlWO, enhancing transcriptional activation of *SlMTR1* and *SlMTR2* and promoting trichome formation (Xie et al., 2022). To the HD‐ZIP subfamily IV belongs also SlCD2 (CUTIN DEFICIENT 2), a regulator of cuticle formation of epidermal cells that positively impacts type VI trichome density (Nadakuduti et al., 2012). Lastly, the HD‐ZIP IV TF SlHD8 (HOMEODOMAIN PROTEIN 8) is involved in trichome types I, III, V, and VI initiation and morphogenesis. SlHD8 regulates *SlHl* (*HAIRLESS*) and its homolog *SlHl‐2*, coding respectively for two interacting subunits of the WAVE (WASP‐family verprolin‐homologous protein) regulatory complex for actin filaments nucleation. SlHD8 function is required for the JA‐induced trichome elongation by enhancing the expression of proteins determining the loosening of the cell wall and is regulated by SlJAZ4 (Hua et al., 2021; Kang et al., 2010; Kang et al., 2016; Xie et al., 2020).

Although in the last decade many studies have led to the discovery of tomato genes involved in type VI glandular trichome development, a comprehensive regulatory model is far from being achieved. In this study, we characterized *glandless*, a tomato mutant where type VI trichomes miss the glandular head cells and do not accumulate volatile terpenes. Additionally, we identified the mutated gene coding for a novel HD‐ZIP TF, named *SlHDZ38*, the first of subfamily I linked to trichome regulation in tomato. With virus‐induced gene silencing (VIGS) we further confirmed that SlHDZ38 is indispensable for the gland development of type VI trichomes. SlHDZ38 is also involved in controlling the densities of other trichome types. Altogether, these results provide new insights in the regulation of type VI glandular trichome development.

## RESULTS

### Phenotype of a new tomato trichome mutant

**Figure 1:**
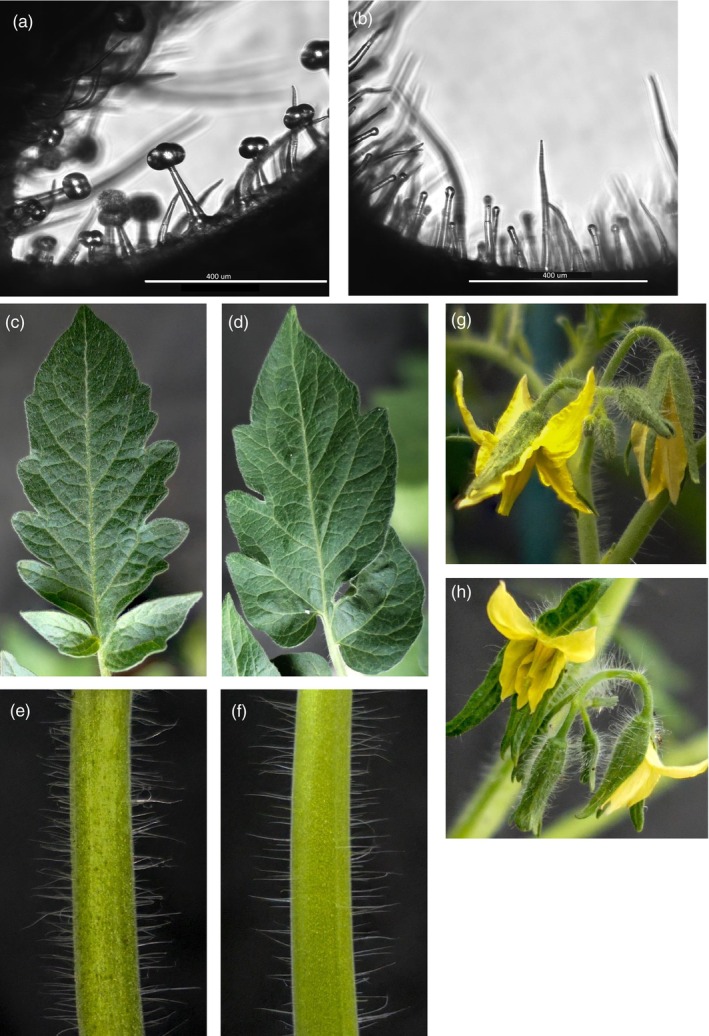
Phenotype of glandless mutant. Type VI glandular trichomes as seen with an EVOS inverted light microscope on leaves of RZ‐2 wild‐type (WT) (a) and on the glandless mutant (b) tomato. Comparison between wild‐type (WT) and glandless leaves (c–d) and trichomes visible on respectively wild‐type (WT) and glandless stems (e–f) and sepals (g–h).

To select tomato plants with aberrant type VI trichomes, the trichomes of 8000 individuals from an EMS‐mutagenized population were phenotyped with a stereo microscope. Previously, in another EMS population, the *myc1* mutant had been identified (Xu et al., 2018). In this study, we identified a mutant with aberrant trichomes missing the glandular cells of type VI glandular trichomes, and for this reason, it was named *glandless* (Figure 1a,b). Visually, the most evident phenotype of *glandless* plants seemed a reduced density of hairy trichomes on leaves (Figure 1c,d), stems (Figure 1e,f) and sepals (Figure 1g,h).

### The *glandless* mutant has aberrant type VI glandular trichomes

**Figure 2:**
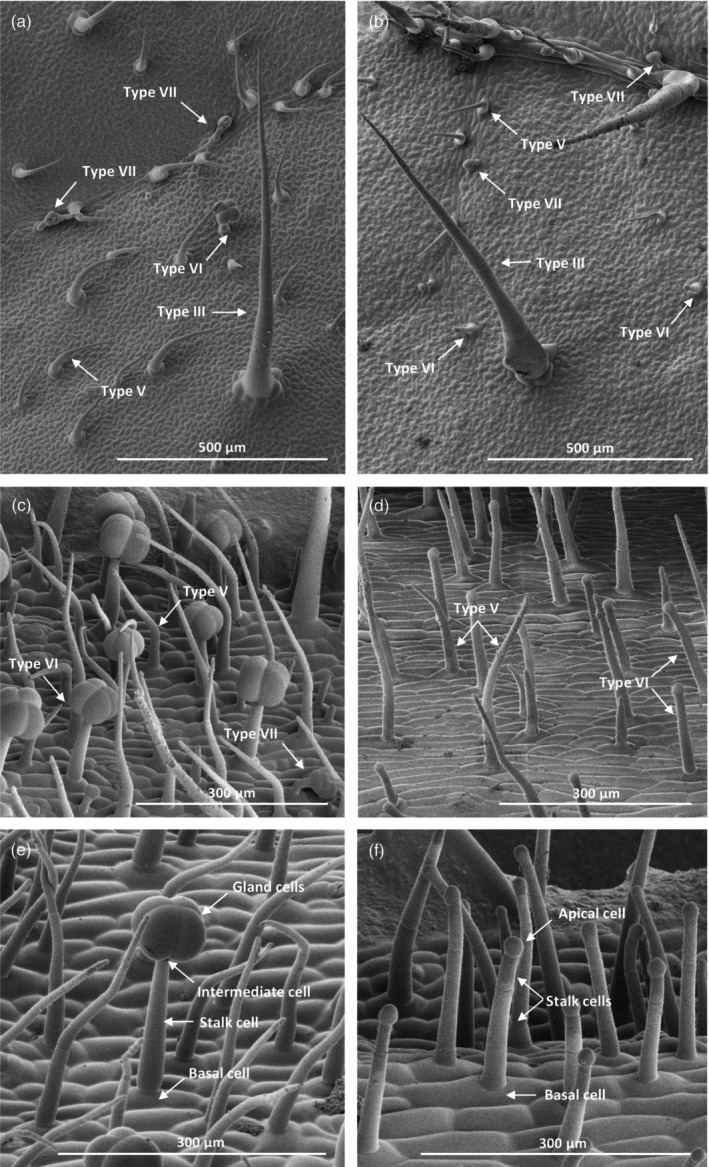
Morphology of glandless trichomes on leaves and stems. Cryo‐SEM images of RZ‐2 wild‐type (WT) leaves (a) and stems (c, e) and glandless mutant (MUT) leaves (b) and stems (d, f). Arrows indicate the different types of trichomes and the cell types that constitute type VI glandular trichomes.

To fully investigate the trichome morphology on the *glandless* mutant plant, leaf (Figure 2a,b) and stem surfaces (Figure 2c,d) were imaged using Cryo‐SEM. Architecture and dimensions of nonglandular hairy trichomes of type III and V were not different between RZ‐2 wild‐type (WT) and *glandless* on both leaf sides and on stems. Likewise, on *glandless* leaves, glandular trichomes of type VII and of the rarely occurring type I were like the WT. Instead, detailed inspection of type VI trichome morphology supported our previous observations as no trichomes resembling the WT type VI (Figure 2e) could be found on the stem and leaves of *glandless* (Figure 2f). The novel trichome type in this mutant emerges from a single simple epidermal basal cell similarly to type VI and has a comparable‐sized stalk supporting, presumably, a single cell. However, the stalk is formed not by one but by two, or sometimes three, cells, suggesting a possible alteration in the cell division pattern. The four gland cells forming type VI trichome head are absent and, in their place, *glandless* type VI trichome heads exhibit a single rounded cell. Comparison with type IV trichomes on cotyledons (Figure S1) indicates similarities between the mutant type and type IV trichomes, which are normally absent from adult leaves (Glas et al., 2012; Vendemiatti et al., 2017). However, the top cells seem to be different from the single gland cell of type IV trichomes (Figure S1). In tomato, the metabolites in the glands of type IV and VI trichomes can be released when physically damaged by a stretched‐out glass Pasteur pipette (Kortbeek et al., 2023; McDowell et al., 2011; Tissier et al., 2017b). However, on *glandless*, the single round apical cell could not be ruptured in this manner, suggesting it to be an intermediate cell rather than a gland cell. Overall, these results suggest that a mutation in *glandless* involves a gene regulating type VI trichome development causing the observed anomalous morphology.

### Leaf trichome densities are different in *glandless* mutant

**Figure 3:**
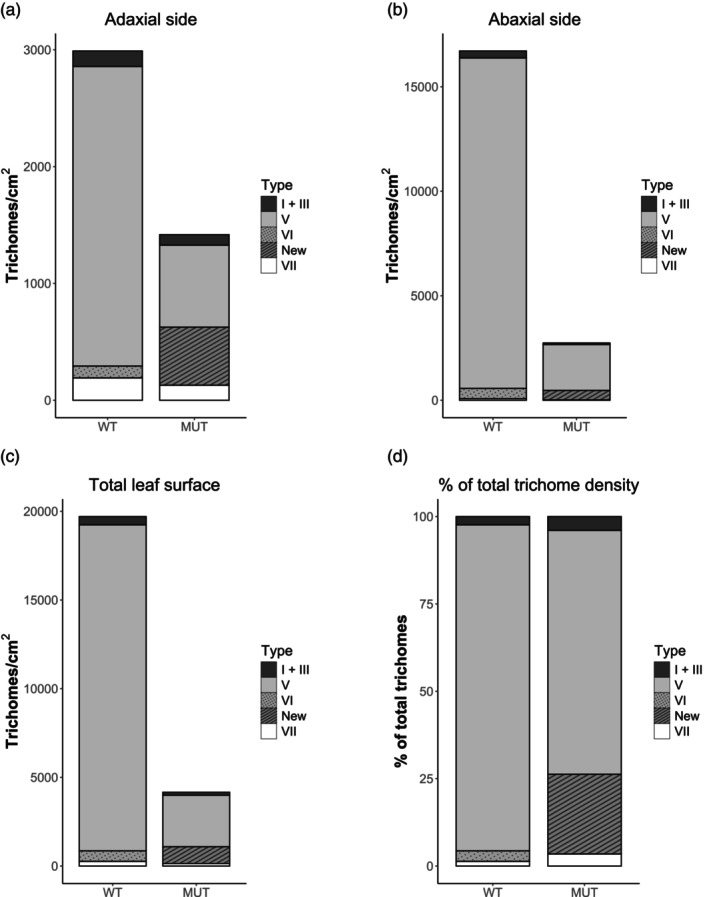
Leaf trichome densities. Densities of type I and III, type V, type VI, type VII, and of the new trichome type on (a) adaxial side, (b) abaxial side, and (c) on total leaf surface of RZ‐2 wild‐type (WT) and glandless mutant (MUT) plants. (d) Percentage of each trichome type of the total leaf trichome density.

To examine whether the *glandless* mutation influences also trichome densities, Cryo‐SEM images of both the adaxial and abaxial leaf surfaces were examined. On both the adaxial (AD) and the abaxial (AB) leaf surfaces, no difference was observed in the number of type I and III trichomes, but significantly fewer type V trichomes were found on both leaf sides (Figure 3a,b; Figure S2). On the AD side, there were significantly more mutant type VI trichomes on *glandless* than normal VI trichomes in the RZ‐2 WT but not on the AB side of the leaves (Figure 3a,b; Figure S2). Instead, type VII glandular trichomes had lower density on the AB but not on the AD side (Figure 3a,b; Figure S1). Overall, the trichome density is much reduced in the *glandless* mutant (Figure 3c) and the number of the different type of trichomes was also changed (Figure 3d). The number of the different types of trichomes on sepals also differed between the mutant and the WT (Figure S3). Altogether, these results indicate that the selected *glandless* line is a new trichome mutant that presents not only altered type VI trichome morphology but also has different type V and type VII densities.

### In the *glandless* mutant the levels of volatile mono‐ and sesquiterpenes are reduced

**Figure 4:**
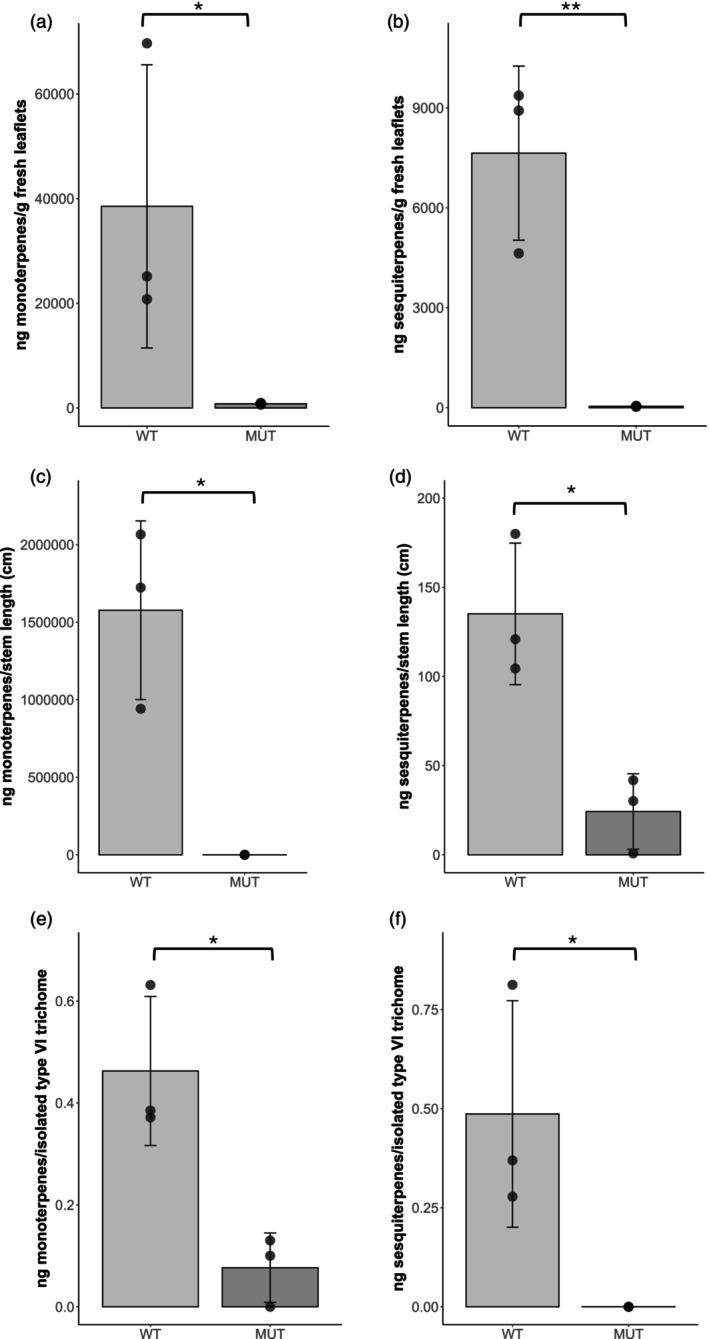
Total known volatile terpenes levels in leaves, stems, and isolated type VI trichomes. Total mono‐(a, c, e) and sesquiterpene (b, d, f) levels respectively, in stems (a, b), leaves (c, d) and isolated type VI trichomes (e, f) of RZ‐2 wild‐type (WT) and glandless mutant (MUT) tomato plants (*n* = 3). The bars represent the mean values ± standard deviation (SD) of the sum of the levels of targeted volatile terpenes quantified by GC–MS and normalized by stem length, leaf fresh weight, or number of isolated type VI trichomes. Differences in bars are annotated accordingly to the significance levels resulting from independent *t*‐tests after Shapiro–Wilk's normality test and *F*‐test comparison of variances (*P < 0.05, **P < 0.01). Significance for the nonnormally distributed subset was assessed with the Wilcoxon nonparametric test.

To study whether the lack of the four glandular cells on the *glandless* mutant also impacted the production of volatile terpenoids synthetized and stored in type VI glandular trichomes, monoterpene (α‐pinene, 2‐carene, α‐phellandrene, α‐terpinene, D‐limonene, β‐phellandrene trans‐β‐ocimene, terpinolene, linalool), and sesquiterpene (β‐caryophyllene and α‐humulene) levels were quantified on RZ‐2 WT and *glandless* mutant (Figure 4 and Figure S4). In the *glandless* mutant, all the measured mono‐ and sesquiterpenes were significantly reduced on both stems (Figure 4a,b) and leaves (Figure 4c,d). Similar results were observed also when analyzing 200 gland cells manually isolated from type VI trichomes on leaves from the RZ‐2 WT and the corresponding cell left on the new trichomes in *glandless* (Figure 4e,f). These results show that the *glandless* mutation affects the levels of volatile mono‐ and sesquiterpenes suggesting either that *glandless* directly regulates terpene biosynthesis or that the reduction is an indirect effect of the alteration of type VI glandular trichome morphology.

### *Glandless* is mutated in Solyc09g008810

**Figure 5:**
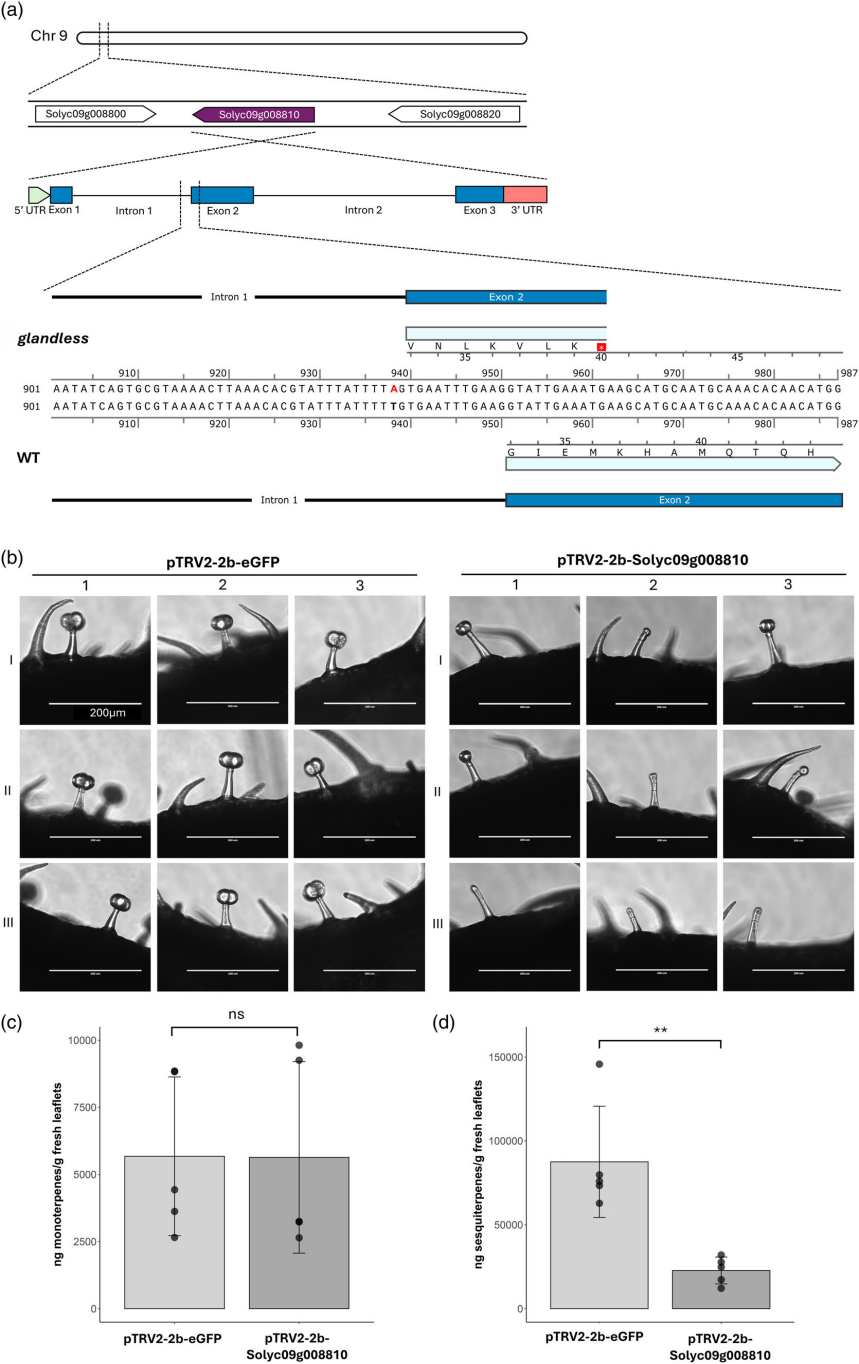
Mapping of glandless mutation and validation of phenotype with VIGS in Micro‐Tom. (a) The SNP was mapped on chromosome 9 in the Solyc09g008810 gene. Compared with the RZ‐2 wild‐type sequence (WT) in glandless the T938A substitution generated a premature splicing acceptor site causing a frameshift and an early stop codon. (b) 5 weeks after VIGS in wild‐type Micro‐Tom tomato plants, targeting Solyc09g008810, or GFP as negative control an aberrant type VI trichome phenotype was observed with a microscope, on 3 different leaves (I‐III) of three independent plants (1–3). Additionally, of the same Micro‐Tom plants the total levels ± standard deviation (SD) of known volatile monoterpenes (c) and sesquiterpenes (d) were quantified with GC–MS (*n* = 4). Differences in bars are annotated accordingly to the significance levels resulting from independent t‐tests after Shapiro–Wilk's normality test and *F*‐test comparison of variances (**P < 0.01, ns nonsignificant).

The *glandless* mutation was fine‐mapped to chromosome 9, specifically to the Solyc09g008810 gene (Figure 5a; Data S1). To determine the mutation, the genomic sequence of this gene in the RZ‐2 WT and in the *glandless* mutant were cloned and sequenced. The *glandless* allele presented a T to A substitution in position 938 near the 3′ end of the first intron. This single‐nucleotide polymorphism (SNP) introduces a premature acceptor splicing site (AG) that causes a 10 bp frameshift to the coding sequence (CDS) of the gene that results in an early stop codon after a stretch of seven amino acids (Figure 5a). To confirm that this mutation is responsible for the observed trichome phenotype, a functional analysis of the Solyc09g008810 gene was performed using VIGS in *S. lycopersicum* cv. Micro‐Tom. We also targeted eGFP as a negative control and *SlPDS1* (phytoene desaturase) as a visible control, as it leads to photobleaching. After 5 weeks, microscopical observations revealed that compared with the negative control, leaves in which Solyc09g008810 was targeted had patches with aberrant type VI glandular trichomes that resembled the morphology of the ones observed on the *glandless* mutant (Figure 5b). Accordingly, the levels of known mono‐ and sesquiterpenes were reduced (Figure 5c). Therefore, we concluded that the Solyc09g008810 gene, carrying the SNP site in the *glandless* mutant, is responsible for the mutant trichome phenotype observed on this line. Overall, these results demonstrate that a mutant allele of the Solyc09g008810 gene is responsible for the trichome phenotypes in the *glandless* tomato mutant, suggesting that the encoded protein controls the development of type VI glandular trichomes.

### SlHDZ38 is a new candidate regulator of tomato trichomes

**Figure 6:**
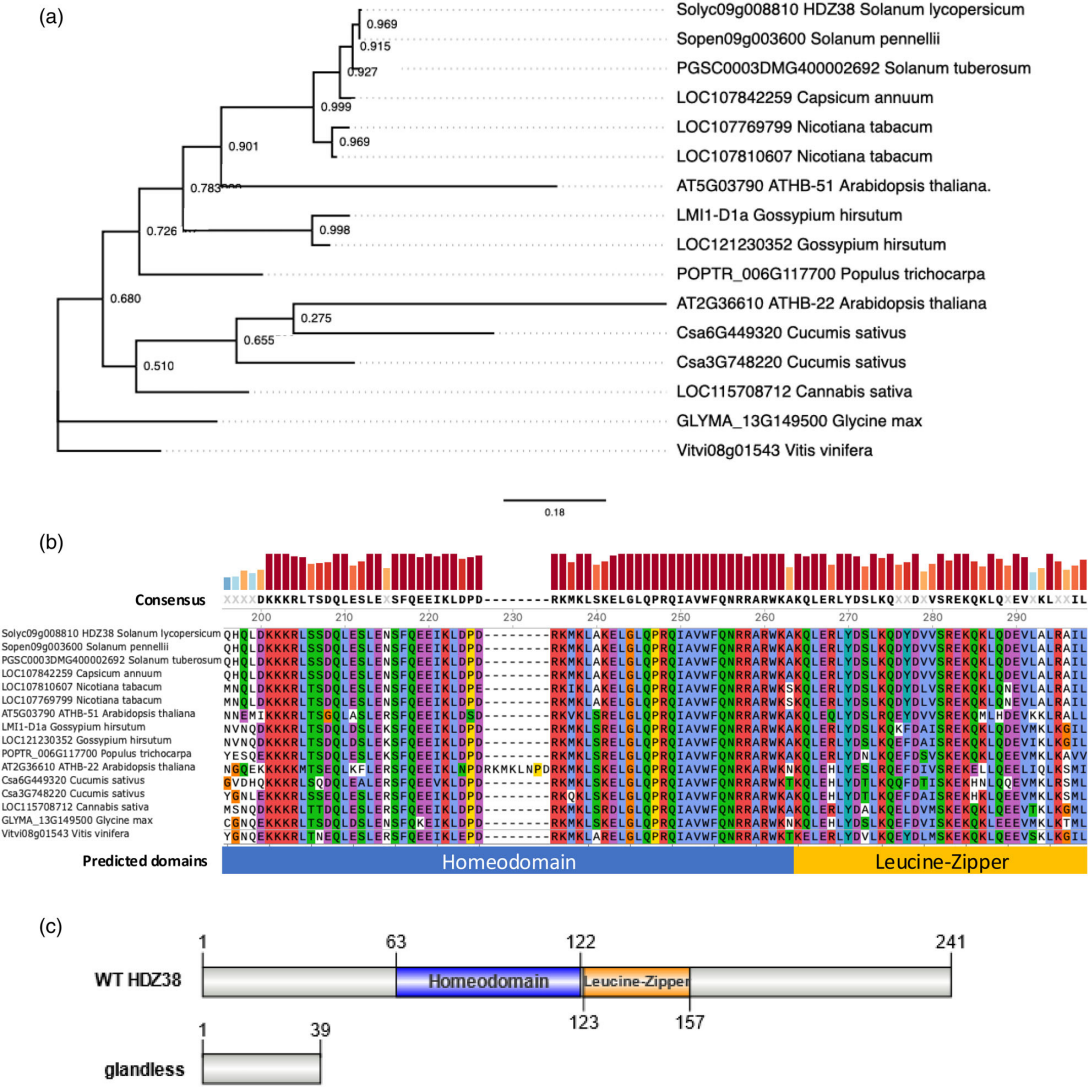
Phylogenetic relationship of SlHDZ38 homologs in selected plants. (a) Putative homologs of SLHDZ38 protein were selected from BLASTp (NCBI) results, aligned with TCoffee algorithm and used to construct a phylogenetic tree in PhyML with the maximum‐likelihood method. Values on the branches indicate the supporting bootstrap values of 500 replications. (b) Detail of the multisequence alignment shows the high conservation of the Homeodomain and the Leucine zipper motifs among selected homologs. (c) The truncated protein encoded by the glandless allele of lacks both the functional domains of the RZ‐2 wild‐type protein (WT).

The Solyc09g008810 gene is predicted to code for a 241 amino acids long uncharacterized protein with a homeobox domain, widely reported to mediate specific binding to DNA, and a leucine zipper motif, that is known to mediate formation of protein dimers. This classifies the protein as part of subfamily I of the plant‐specific HD‐ZIP TF family. In tomato, a total of 49 genes belong to it (Hong et al., 2021) and Solyc09g008810 gene encodes SlHDZ38. To gain more insight in its functions, the amino acid sequence of SlHDZ38 was used to find homologs via BLASTp on the NCBI nonredundant protein database. The homology research returned many HD‐ZIP‐I TFs from various plant species, including hemp, grapevine, poplar, cotton, and cucumber (Figure 6a). The alignment of the selected plant homologs of SlHDZ38 revealed a high level of conservation among HD‐ZIP I transcription factors, particularly in the regions of the homeobox domain (Q63‐A122) and the leucine zipper motif (K123‐L157) (Figure 6b). The closest homolog in *Arabidopsis thaliana* is ATHB51 (HOMEOBOX 51) also known as ATLMI1 (LATE MERISTEM IDENTITY 1). Remarkably, in *Cucumis sativus*, a TF characterized as a regulator of trichome differentiation was found, encoded by the CsGL1 (GLABROUS 1; Csa3G748220) gene, whose mutant alleles named Cs*GL1*, Cs*MICT* (*MICRO‐TRICHOME*), and Cs*TBH* (*TINY BRANCHED HAIR*) all show a glabrous phenotype (Q. Li et al., 2015; Pan et al., 2021; Zhang et al., 2021; Zhao et al., 2015). The *glandless* allele, due to an early stop codon, encodes for a 39 amino acid long protein that completely lacks both the DNA‐binding and the dimerization domains (Figure 6c). These results corroborate our finding that the *glandless* phenotype is caused by the mutation in SlHDZ38.

### SlHDZ38 *glandless* mutation alters the expression of trichome‐specific regulatory and metabolic genes

With the aim to study the impact of the mutation at the transcriptional level, RNA sequencing was performed on leaves, including their trichomes, and isolated stem trichomes of RZ‐2 WT and *glandless* plants (Table S2). The expression of genes already known to be involved in trichome development and in type VI trichome‐specific metabolism was analyzed. In leaves, seven mono‐ and sesquiterpene synthase genes were less expressed (Table 1). Additionally, reduced expression was found for the JA signaling inhibitors SlJAZ2 and SlJAZ4 that control SlMYC1 and its interaction with SlWO, essential in the regulation of type VI trichome development and terpene metabolism (Hua et al., 2020; Xu et al., 2018). Slightly but significantly lower transcript levels in *glandless* leaves were also observed for SlMTR1 and two HD‐ZIP TFs, SlLN, and SlHD8, all three involved in trichome development. SlGCR1, one of the two recently identified MYB‐like TFs that inhibit the formation of trichome gland cells (Chang et al., 2024) was also found to have lower expression levels in *glandless*. These results support the double role for SlHDZ38 as being involved – directly or indirectly by modulating the levels of other TFs – in controlling volatile mono‐ and sesquiterpenes biosynthesis and regulating trichome development.

**Table 1:** Differentially expressed trichome genes in leaves of *glandless* mutant

Solyc ID	Gene	log2FC	*P*‐value	Class	Family	Annotation
Solyc06g059930	TPS9	−10.34	2.9E‐15		TPS	Sesquiterpene synthase (Germacren), trichome‐specific, cytosol
Solyc08g005710	TPS41	−7.91	7.9E‐12		TPS	Terpene synthase, mitochondrial
Solyc01g105850	TPS1	−6.51	2.6E‐05		TPS	Monoterpene synthase (Camphene/tricyclene), chloroplastic
Solyc10g005390	TPS39	−5.19	3.4E‐02		TPS	Monoterpene synthase (Linalool/nerolidol), cytosolic
Solyc12g006570	TPS17	−4.65	3.2E‐06		TPS	Sesquiterpene synthase (Valencene/bisabolene), cytosolic
Solyc08g005670	TPS10	−2.94	3.7E‐02		TPS	Monoterpene synthase (Phellandrene), cytosolic
Solyc03g006550	TPS46	−2.40	2.2E‐09		TPS	Putative geranyllinalool synthase
Solyc08g005720	TPS18	−2.27	3.2E‐03		TPS	Terpene synthase, mitochondrial
Solyc12g049400	JAZ4	−2.55	7.0E‐13	TF	Tify	Jasmonate ZIM‐domain protein, Tify
Solyc12g009220	JAZ2	−1.20	1.3E‐06	TF	Tify	Jasmonate ZIM‐domain protein 1
Solyc02g076670	GCR1	−1.03	6.7E‐06	TF	GARP‐G2‐like	Myb domain, Homeodomain‐like protein
Solyc10g083140	MTR1	−0.77	1.5E‐03		Cyclin	Hypothetical protein
Solyc03g031760	LN	−0.64	3.6E‐05	TF	HD‐ZIP	Homeobox‐leucine zipper protein ROC2
Solyc03g098200	HDZ8	−0.56	2.7E‐02	TF	HD‐ZIP	Homeobox‐leucine zipper protein HDG12

Many terpene synthases were among the lowest expressed genes also in isolated stem trichomes, including the type VI glandular trichome‐specific *SlTPS9* and *SlTPS5* (Table 2). Dramatically reduced were also the transcriptional levels of SlEOT1, SlEOT2, SlMYC1, and SlSCL3, that share a particularly high expression in trichomes and function as transcriptional regulators of terpene biosynthetic pathway genes (Spyropoulou, 2012; Spyropoulou et al., 2014a, 2014b; Xu et al., 2018; Xu, 2023; Yang et al., 2021). In the isolated stem trichomes of *glandless*, among factors known to be involved in trichome development, SlSH, SlMIXTA‐like and the HD‐ZIP IV TFs SlHD8 and SlCD2 had higher transcript levels. Moreover, *SlWO* and two of its downstream targets in hairy trichome regulation, *SlMX1*, and *SlWOX3b*, were all upregulated. Accordingly, *SlMTR1* a negative regulator *of SlWO* showed lower expression, while its homologs *SlMTR2* and *SlMTR3* had higher expression. Lastly, the JA signaling inhibitors *SlJAZ5*, *SlJAZ6*, and *SlJAZ7* were also more expressed in *glandless* than in WT.

**Table 2:** Differentially expressed trichome genes in isolated stem‐trichomes of *glandless* mutant

Solyc ID	Gene	log2FC	*P*‐value	Class	Family	Annotation
Solyc10g005390	TPS39	−8.66	2.90E‐14		TPS	Monoterpene synthase (Linalool/nerolidol), cytosolic
Solyc08g005720	TPS18	−8.64	1.72E‐17		TPS	Terpene synthase, mitochondrial
Solyc01g105850	TPS1	−8.41	5.65E‐13		TPS	Monoterpene synthase (Camphene/tricyclene), chloroplastic
Solyc08g005670	TPS10	−8.41	7.85E‐10		TPS	Monoterpene synthase (Phellandrene), cytosolic
Solyc01g101170	TPS31	−8.38	4.20E‐12		TPS	Sesquiterpene synthase (Viridiflorene), cytosolic
Solyc06g059930	TPS9	−8.38	6.90E‐08		TPS	Sesquiterpene synthase (Germacren), trichome‐specific, cytosol
Solyc07g008690	TPS16	−8.28	3.93E‐20		TPS	Sesquiterpene synthase
Solyc08g005710	TPS41	−8.02	1.85E‐13		TPS	Terpene synthase, mitochondrial
Solyc08g005640	TPS21	−5.60	5.40E‐65		TPS	Terpene synthase, plastidic
Solyc01g105890	TPS5	−5.52	7.16E‐17		TPS	Monoterpene synthase (Linalool), trichome‐specific, plastidic
Solyc01g101210	TPS35	−5.35	8.40E‐03		TPS	Terpene synthase, cytosolic
Solyc01g105920	TPS7	−2.90	1.49E‐04		TPS	Monoterpene synthase (Beta myrcene/limonene), plastidic
Solyc12g006570	TPS17	−1.71	2.67E‐10		TPS	Sesquiterpene synthase (Valencene/bisabolene), cytosolic
Solyc02g062400	EOT1	−3.96	2.92E‐79	TF	SRS	Expression of terpenoids 1
Solyc08g005050	MYC1	−3.66	8.94E‐55	TF	bHLH	bHLH Transcription factor MYC1
Solyc03g033680	EOT2	−2.45	1.60E‐21	TF	SRS	Expression of terpenoids 2
Solyc12g099900	SCL3	−2.08	5.06E‐17	TF	GRAS	Scarecrow‐like 3
Solyc09g008810	HDZ38	−1.56	5.36E‐04	TF	HB‐HD‐ZIP	Homeobox‐leucine zipper protein ATHB‐22
Solyc10g083140	MTR1	−0.56	8.48E‐03		Cyclin	hypothetical protein
Solyc09g014980	DT1	0.34	4.72E‐03	TF	SCAR	Protein SCAR4
Solyc03g098200	HDZ8	0.41	1.15E‐03	TF	HB‐HD‐ZIP	Homeobox‐leucine zipper protein HDG12
Solyc01g091630	CD2	0.52	8.96E‐07	TF	HB‐HD‐ZIP	Cutin deficient 2
Solyc03g118540	JAZ5	0.66	4.43E‐02	TF	Tify	Jasmonate ZIM domain protein
Solyc02g088190	MIXTA‐like	0.71	4.88E‐07	TF	MYB	MYB transcription factor
Solyc02g076670	GCR1	0.72	9.43E‐05	TF	GARP‐G2‐like	Myb domain‐, Homeodomain‐like protein
Solyc01g005440	JAZ6	0.99	3.38E‐07	TF	Tify	Jasmonate ZIM‐domain protein
Solyc06g073990	MTR2	0.99	7.18E‐06		Cyclin	Hypothetical protein
Solyc02g080260	WO	1.01	2.49E‐11	TF	HB‐HD‐ZIP	Woolly
Solyc01g007870	MTR3	1.09	1.88E‐07		Cyclin	hypothetical protein
Solyc11g011030	JAZ7	1.11	5.60E‐03	TF	Tify	Pto‐responsive gene 1
Solyc01g010910	MX1	1.21	3.45E‐03	TF	MYB	MYB transcription factor subfamily 9
Solyc11g072790	WOX3b	1.90	1.67E‐04	TF	HB‐WOX	WOX3b
Solyc10g078990	SH	4.17	4.23E‐04	TF	C2H2	Zinc finger protein 6

Very interestingly, a set of genes related to acylsugar biosynthesis was upregulated in the *glandless* mutant (Table S3). This made us investigate the possibility that acylsugar biosynthesis had increased in the *glandless* mutant. Using leaf dips, we indeed measured higher acylsucrose levels in the *glandless* mutant than in the corresponding WT (Figure S5, Table S4). These acylsucrose levels were much lower than in *S. pennellii*, and no acylglucose molecules were detected as has been previously shown (Mutschler et al., 1996; Steffens & Walters, 1991). To determine whether these acylsugars were made in the new type trichomes we performed a Rhodamine B staining (Lin & Wagner, 1994; Vendemiatti et al., 2017). Type IV trichomes on the cotyledons of the *glandless* mutant and the corresponding RZ‐2 WT were indeed stained by Rhodamine B (Figure S6). However, neither the new type trichomes on *glandless* leaves nor the type VI trichomes on the WT were stained. This implies that these new trichomes do not accumulate acylsugars in their apical cells.

Altogether, the simultaneous differential expression of trichome‐specific metabolic enzymes and regulatory transcription factors in isolated stem trichomes represents another piece of evidence that SlHDZ38 is involved in trichome development and specialized metabolism.

## DISCUSSION

This study revealed that a mutant allele of SlHDZ38, a novel HD‐ZIP subfamily I transcription factor, is responsible for the *glandless* tomato mutant phenotype. We demonstrated that HDZ38 is essential for the development of type VI glandular trichomes and that it is also involved in the regulation of the densities of type VII glandular and type V nonglandular trichomes. Additionally, we showed that SlHDZ38 is relevant for the production of certain specialized metabolites.

### SlHDZ38 is a new regulator of type VI glandular trichome development

Tomato has become the model plant to study the regulation of development and specialized metabolism of multicellular glandular trichomes in horticultural crops. Among the proteins characterized so far as regulators of these two processes, some belong to the subfamily IV HD‐ZIP TF family, such as SlWO and SlCD2. In this study, we characterized a novel tomato mutant, *glandless*, showing a new type of trichome but missing type VI glandular trichomes. By mapping the mutation, we found that a subfamily I HD‐ZIP TF, SlHDZ38, underlies the *glandless* mutation, as verified by VIGS that generated an identical mutant trichome phenotype. Overall, these results demonstrate that SlHDZ38 is a regulator of type VI glandular trichome development, specifically of the glandular head formation.

The cucumber homolog of HDZ38, CsGL1, whose mutant exhibits aborted glandular trichome development, is expressed during the developmental stage where the glandular head formation occurs (Dong et al., 2022), supporting our conclusion of a similar role for SlHDZ38 in tomato. Many TFs of the HD‐ZIP subfamily I, including SlHB2 in tomato, have been previously shown to regulate auxin signaling and transport, and developmental responses to light, drought, salt, cold, and heavy metal stresses (Gong et al., 2019; Hu et al., 2017). However, SlHDZ38 of this subfamily clearly has a role in glandular trichome development.

The absence of mono‐ and sesquiterpenes in *glandless* plants and the upregulation of acylsugar genes and biosynthesis lead us to the following hypothesis: the new type of trichome in the *glandless* mutant is either an incomplete type IV or type VI trichome. In both cases, it expresses acylsucrose biosynthesis genes. These trichomes are incomplete as there is no gland cell on top since no metabolites can be harvested when touched by a glass capillary. Still, precursors of acyl sugars, sucrose, and fatty acids can be transported through the trichome stalk to the apical cell where they are enzymatically converted into acylsugars.

### SlHDZ38 is part of the tomato trichome regulatory network

Many TFs that are normally highly expressed in gland cells of type VI trichomes have lower transcript levels in isolated stem trichomes of *glandless* when compared with WT. Some of these are involved in trichome development such as SlMTR1 and SlHD8, whereas others participate in regulating specialized metabolism, such as EOT1 and EOT2, or both processes, such as SlMYC1 and SlSCL3. In leaves the situation is somewhat peculiar: although *SlHDZ38* is hardly expressed in WT leaf tissue—that includes trichomes—the *glandless* mutation still reduces the expression of *SlMTR1* and *SlHD8*, genes known to be involved in trichome development. To determine whether these genes are regulated directly by SlHDZ38 one could perform chromatin immunoprecipitation followed by sequencing (ChIP‐seq; Boersma et al., 2022). This would establish whether regulation of the specialized pathways is direct or a consequence of aberrant trichome development. It would also resolve whether SlHDZ38 regulates both trichome development and specialized metabolism as previously suggested for SlMYC1 (Xu et al., 2018).

It has been recently suggested (Wu et al., 2023) that higher SlWO levels preferentially activate *SlMX1* and *SlWOX3* expression, promoting the differentiation of type I, II, III, IV, and V trichomes—by Wu referred to as digitate trichomes—and inhibiting the formation of type VI and VII trichomes—by Wu referred to as peltate trichomes. In isolated stem trichomes of the *glandless* mutant, the transcript levels of these three TFs are all higher, suggesting that SlHDZ38 regulates them, directly or indirectly. Interestingly, the expression of *SlWO*, *SlMX1*, and *SlWOX3* are unaltered in *glandless* leaves with trichomes. Nevertheless, the densities of type V and VII trichomes were severely reduced on *glandless* leaves (Figure S2). Conversely, the densities of the aberrant type VI glandular trichomes were higher on the *glandless* leaves. Therefore, SlHDZ38 could promote the formation of type VI as well as the densities of type V and VII in WT leaves without involving the WO‐WOX3b‐MX1 module. Previously, we have shown that type VI glandular trichomes are differently regulated on leaves and stems by SlMYC1 (Xu et al., 2018). It could be that the regulatory modules controlling trichome development and densities in leaf and stem trichomes are indeed different. Since null mutants of *Wo* are unable to form most trichome types (Wu et al., 2023), Wo might be acting upstream of SlHDZ38 in leaves. Since transcript levels of *Wo* are higher in the stem trichomes of the *glandless* mutant, there might be another level of fine‐tuning of the SlWO‐dose‐dependent regulatory mechanism. Alternatively, it could indicate that the concentration of WO protein is mostly controlled at the post‐transcriptional level and that the transcriptional regulation by SlHDZ38 may have a different or later role.

To be further investigated is also the interplay between JA signaling and SlHDZ38. It has been shown that JA can regulate tomato trichome initiation, development, and also specialized metabolites biosynthesis (Boughton et al., 2005; Hua et al., 2021; Li et al., 2004; Xu et al., 2018; Yan et al., 2013; Yang et al., 2021; Yoshida et al., 2009). Previous studies suggested that SlJAZ2 inhibits the functionality of the SlWO‐SlMYC1 module in activating terpenes biosynthesis in type VI glandular trichomes, via a competitive binding mechanism (Hua et al., 2020). Additionally, the overexpression of SlJAZ2 caused downregulation of SlWO and SlMTR1 in stem epidermis and decreased the abundance of stem trichomes (Yu et al., 2018). SlJAZ4, which shows its highest expression in trichomes, inhibits via protein–protein interaction the activity of SlMYC1 as activator of terpene biosynthesis (Hua et al., 2020) and of SlHD8 responsible of JA‐induced trichome elongation (Hua et al., 2021). Accordingly, SlJAZ4 overexpression resulted in shorter trichomes (Hua et al., 2021). The differential gene expression analysis for the *glandless* mutant showed that in leaves, genes coding for two JA signaling inhibitors, SlJAZ2, and SlJAZ4 have lower expression than in WT plants. This suggests that SlHDZ38 maintains *SlJAZ2* and *SlJAZ4* expression levels in the absence of JA, consequently contributing to stabilization of SlWO and SlMTR1 levels and hence trichome initiation, and to preserve the inhibition of SlHD8 and the SlWO‐SlMYC1 module activity. Interestingly, in stem trichomes, no difference was observed for SlJAZ2 and SlJAZ4 levels but SlJAZ5, SlJAZ6, and SlJAZ7 were higher expressed than in the WT. This suggests a different role for SlHDZ38 related to JA signaling in stem trichomes, where it seems to act as negative regulator of SlJAZ5, SlJAZ6, and SlJAZ7.

Overall, our results point once more to the very intricate regulatory network of TFs that determines the developmental fate of trichomes in tomato and the regulation of their specialized metabolism. Accordingly, it remains difficult to determine the precise role of SlHDZ38 in this network, and more detailed studies focusing on protein–protein interactions with other known TFs or on binding and activation of their promoters are necessary for this goal. We suggest it to be located high up in the hierarchy in this network, as it appears from the differential expression of other master regulators. To further expand our comprehension of the role of SlHDZ38 in the trichome regulatory network and validate some of the suggested hypotheses, further studies should also investigate the effect of *SlHDZ38* overexpression on the regulatory network of TFs and on trichome densities and morphology.

## EXPERIMENTAL PROCEDURES

### Plant material and growing conditions

Tomato (*Solanum lycopersicum*) cv. Micro‐Tom plants were germinated and grown in soil at 16/8 h and 23/18°C day/night conditions in a greenhouse, supplemented when necessary with artificial light (150 mE m^−2^ s^−1^; Philips Master Green Power). The second and third couple of leaflets (counting from the leaf tip) of the fourth true leaf (counting from the plant top) of 4‐week‐old tomato plants were used for all the experiments, unless otherwise specified. *In vitro* tissue and plant cultures were grown in a growth chamber (24°C, 70%RH, 16 h light/8 h dark) on ½ strength Murashige and Skoog (MS) medium (MS basal salts with vitamins 2.2 g/L, sucrose 5 g/L, MES 1 g/L, 0.8% agar, pH 5.8 with KOH) occasionally with the addition of the required supplements. WT tomato breeding line Rijk Zwaan (RZ)‐2 was mutagenized by submerging at room temperature for 24 h, 10 000 seeds in an aerated solution of 0.5% (w/v) ethyl methanesulfonate (EMS). The plants of these seeds were grown in a greenhouse to produce M2 seeds. M2 seeds were harvested and bulked in one pool used to screen 8000 plants with a stereomicroscope to identify individuals exhibiting an aberrant type VI glandular trichome phenotype. By fine mapping of the BC_1_M_2_ population obtained by backcrossing of the selected mutant line, the recessive trait was localized on chromosome 9, specifically to the Solyc09g008810 gene. M6 seeds from the same selected mutants were used for all the phenotyping experiments.

### Constructs generation

For the VIGS of the *SlHDZ38* gene (Solyc09g008810) (Hong et al., 2021), a 300 bp fragment of its cDNA sequence was selected via the Sol Genomics Network (SGN; https://solgenomics.net) VIGS tool (Fernandez‐Pozo et al., 2015), synthesized with the addition at the 5' and 3' ends of attL1 and attL2 gateway sites, respectively (Gene Universal Inc., Newark, DE, USA, www.geneuniversal.com) and recombined via Gateway^®^ LR clonase^®^ II in pTRV2‐2b vector (Valentine et al., 2004) producing pTRV2‐2b‐Solyc09g008810.

### Virus‐induced gene silencing

For the VIGS assay (Liu et al., 2002), the tobacco rattle virus vector pTRV‐2b‐HDZ38 was transformed into *A. tumefaciens* GV3101 (pMP90) by electroporation. A single positive colony was grown overnight (28°C, 200 rpm) in liquid LB medium and resuspended in infiltration buffer (MS basal salts without vitamis 4.44 g/L, sucrose 20 g/L, 10 mM MES, acetosyringone 200 μm, pH 5.6 with NaOH) and left at room temperature for >3 h. An *A. tumefaciens* strain harboring a pTRV‐2b vector targeting the tomato phytoene desaturase gene *SlPDS1* (Solyc03g123760) was used as control for the silencing. As negative control, an *A. tumefaciens* strain carrying a TRV2‐2b targeting the eGFP gene was used. The three strains, respectively mixed in a 1:1 (v/v) ratio with another *A. tumefaciens* strain with the same OD_600_ carrying the tobacco rattle virus vector pTRV1 (Liu et al., 2002) were infiltrated with a needless syringe in cotyledons of 10‐day‐old tomato plants. Leaflets were collected from the plants 4–6 weeks after infiltration and used for microscopy, metabolomics, and expression analyses.

### RT quantitative PCR analyses

To isolate leaf RNA, the second pair of leaflets of the fourth true leaf counting from the bottom of 4‐week‐old plants were collected and immediately frozen in liquid nitrogen. To isolate trichome RNA, stems and petioles of 4‐week‐old whole plants were collected in 50 ml tubes, frozen and shaken in liquid nitrogen with a vortex mixer. Total RNA was extracted using Trizol reagent (Invitrogen™, Carlsbad, CA, USA) and isolated by treatment with TURBO DNase kit (Ambion™, Austin, TX, USA; www.thermofisher.com) to remove DNA. Quantity and quality of RNA was determined respectively with a NanoDrop (Thermo Scientific™, Waltham, MA, USA; www.thermofisher.com) and by electrophoresis on 1% agarose gel. The synthesis of cDNA was performed with RevertAid H Minus Reverse Transcriptase (Thermo Scientific™) starting from 1 to 2 mg RNA. As a control for genomic DNA contaminations a sample with no RT enzyme was included. Quantitative PCRs (qPCR) were performed in 10 ml reactions mixes containing 0.5 ml of cDNA (0.5–2 ng total RNA equivalent), 2 ml HOT FIREPol EvaGreen qPCR Mix Plus (Solis Biodyne; https://solisbiodyne.com) and 4 ml 300 nM of each primer (Table S1) using a QuantStudio™ 3 Real‐Time PCR System (Applied Biosystems™, Foster City, CA, USA, www.thermofisher.com) and the following cycling program: 2 min 50°C, 10 min 95°C, 40 cycles of 15 s 95°C and 1 min 60°C. Primer pairs efficiency was calculated by analyzing a range of cDNA serial dilutions while a sample with no cDNA was included as control for primer dimers. Two technical replicates of three independent biological samples were analyzed. To calculate relative normalized expression levels, primers for the Actin housekeeping gene SlACT7 (Solyc03g078400) were used (Table S1).

### Analyses of volatile terpenes

To analyze volatile terpenes in leaf trichomes, a leaflet of the second pair from the fourth true leaf counting from the bottom of 4‐week‐old plants was collected (*n* = 3–4), weighted and briefly washed (~ 5 s) with 750 ml or 1 ml cold n‐hexane containing 0.5 ng/ml of either benzyl acetate or 1,2,3,4‐Tetrahydronaphthalene (Sigma‐Aldrich, St. Louis, MO, USA; www.sigmaaldrich.com) as internal standard. For stem trichomes, a segment of 7 cm of the stem between the fourth and the fifth internode was sampled and briefly washed (~ 5 s) with 1 ml cold n‐hexane containing 0.5 ng/ml internal standard. To quantify volatile terpenes in isolated type VI trichomes, 200 glandular head were manually collected under stereomicroscope with a stretched glass Pasteur pipette that was rinsed every 20 trichomes into 150 ml cold n‐hexane containing 0.5 ng/ml internal standard. To remove residues of water from all types of extracts, ~ 10 mg Na_2_SO_4_ (Sigma‐Aldrich) was added to the sample. After vortexing for ~10 s, the extracts were centrifuged at 16 200 ***g*** for 5 min and the upper hexane layer was transferred in glass vials vented with N_2_ to prevent oxidation, and stored at −20°C. An Agilent 7890A gas chromatograph coupled with an Agilent 7200 accurate‐mass quadrupole time‐of‐flight (TOF) mass spectrometer (Agilent, Santa Clara, CA, USA; www.agilent.com) were used for the gas chromatography mass spectrometry analysis. And 1 ml of each sample was injected, heated to 275°C and separated on a HP‐5 ms capillary column (30 m × 250 mm; 0.25 mm thick; Agilent), using helium gas as carrier (7.0699 psi; 1 ml/min flow rate). After 3 mins at 40°C the column oven temperature was increased first by 5°C/min until 140°C, then by 10°C/min up to 250°C and then kept at this temperature for 5 min. Ionization was done with electron‐impact (EI) mode at 70 eV under vacuum. With a solvent delay of 4.1 min, detection of 30–350 mu ions was achieved with 50 scans/second. MassHunter Qualitative Analysis software package (Agilent) was used for chromatogram peaks detection and deconvolution with 50 ppm accuracy. For identification and quantification, a mix of terpenes analytical standards, was injected in serial dilutions. Base‐peak ion integration was used and the areas were normalized by internal standard, dilution volume and fresh‐leaflet weight.

### Microscopy

To observe and image trichome phenotype on leaves and stems, a stereomicroscope and an EVOS fl (Life Technologies, Foster City, CA, USA) inverted microscope were used. From 4‐week‐old plants, leaf disks of 0.6 cm diameter (*n* = 3) were taken from the second pair of leaflets of the fourth true leaf. Stem trichomes were observed on 1 cm long longitudinal sections of the main stem, and the 4th‐leaf petiole was taken right above the fourth internode. To study the morphology and density of trichomes with cryogenic scanning electron microscopy (Cryo‐SEM), micrographs were taken at the Electron Microscopy Center at Wageningen University (http://www.wur.nl). Leaf and stem explants were taken from 4‐week‐old plants and mounted on a brass sample holder using a thin layer of Tissue‐Tek compound (EMS, Washington, PA, USA). Samples were frozen by plunging them into liquid nitrogen and subsequently placed in a cryopreparation chamber (MED 020/VCT 100; Leica, Vienna, Austria). To sublimate any water vapor contamination (ice) from the surface, the samples were kept for 3 min at −93°C at 2 × 10^−6^ mbar. Samples were then sputter coated with a 12 nm layer of tungsten and transferred under vacuum to the field emission scanning electron microscope (Magellan 400; FEI, Eindhoven, the Netherlands) onto the sample stage at −120°C. The images were taken with the secondary electron detector set at 2 kV, 13 pA, working distance 4 mm. All images were recorded digitally with FEI maps software: the leaflet disk single images were taken at a scan rate of 100 s (full frame) with image size of 1536 × 1024 8 bit and then stitched together by the software; the stem sections were taken at a scan rate of 100 s (full frame) with image size of 1536 × 1024 8 bit. Quantitative and morphological analysis of the trichomes was performed using Image J software (Schneider et al., 2012).

### Rhodamine B staining of acylsugar content

The acylsugars in individual trichomes were visualized by dipping leaves in a 0.1% (m/v) solution of Rhodamine B in water as described in Lin and Wagner (1994). Stained trichomes were observed using an EVOS fl (Life Technologies) inverted microscope with the Texas Red filter (560/630 nm excitation/emission).

### RNA‐seq analysis in leaf and isolated stem trichomes

RNA was extracted from frozen‐powdered leaves and isolated stem trichomes using NucleoSpin^®^ RNA XS kit (MACHEREY‐NAGEL, Düren, Nordrhein‑Westfalen, Germany; https://www.mn‐net.com). Starting with 500 ng RNA, a poly‐A enrichment was performed with NEBNext Poly(A) mRNA Magnetic Isolation Module (New England BioLabs, Ipswich, MA, USA). Next, the NEBNext Ultra II Directional RNA Library Prep Kit and NEBNext Multiplex Oligos (New England BioLabs) were used to generate the RNA‐seq libraries according to the manufacturer protocol. The libraries were then assessed for size distribution using a 2200 TapeStation System with Agilent D1000 ScreenTapes (Agilent Technologies) and quantified on a QuantStudio 3 Real‐Time PCR System (Thermo Fisher Scientific) with the NEBNext Library Quant Kit (New England BioLabs). The single end sequencing (1 × 75 bp) of the clustered libraries was performed with NextSeq 500/550 High Output Kit v2.5 (75 Cycles) (Illumina, San Diego, CA, USA) on a NextSeq 550 Sequencing System (Illumina). Via a Snakemake pipeline (Bliek et al., 2021), the reads where first quality‐checked and trimmed using fastp (Chen et al., 2018) with default settings, then mapped with STAR (Dobin et al., 2013) on SL4.0 tomato genome (https://solgenomics.net) with maximum 10 multimappers and four mismatches. Finally, read count tables for downstream analyses were generated with featureCounts and annotated according ITAG4.1 SGN annotation (https://solgenomics.net). The differential expressed genes (DEG) were identified using the DEseq2 package (Love et al., 2014) setting the threshold for the log2 fold change >1 and for the Wald test *P*‐value <0.05. Gene ontology enrichment analysis was performed using AgriGO (T. Tian et al., 2017), or together with KEGG pathway enrichment via ShinyGO (Ge et al., 2020; Kanehisa et al., 2021), while STRING was used to predict and visualize putative protein networks (Szklarczyk et al., 2023).

## AUTHOR CONTRIBUTIONS

PZ carried out the experiments, ZvH identified the glandless mutant, AR and MV fine‐mapped the corresponding gene, PZ, RS, and MH designed the research. JG and MU did the acylsugar measurements, EvD did the SEMs and Rhodamine B staining. PZ, RS, and MH wrote and edited the manuscript.

## CONFLICT OF INTEREST

The authors declare that they have no conflicts of interest associated with this work.

## Supporting information


**Table S1.** Primers used in this study.

**Table S2.** Differentially expressed genes in isolated stem trichomes of *glandless* mutant.

**Table S3.** Differentially expressed acylsugar genes in isolated stem trichomes of *glandless* mutant.

**Table S4.** Determination of acylsugars with LC–MS.

**Figure S1.** Close‐up images of different types of trichomes on leaves and stems.
**Figure S2.** Leaf trichome densities.
**Figure S3.** Sepal trichome densities.
**Figure S4.** Volatile mono‐ and sesquiterpenes in leaves, stems, and isolated type VI trichomes.
**Figure S5.** Acylsugar levels on leaves.
**Figure S6.** Rhodamine B staining of trichomes.
**Figure S7.** Multiple sequence alignment of SlHDZ38 (Solyc09g008810) gDNA.
**Figure S8.** Sequence alignment of SlHDZ38 (Solyc09g008810) cDNA.

**Data S1.** Supplemental methods: Mapping of the *glandless* mutation; acylsugar measurements.

## Data Availability

RNA sequencing data used in this study are available at the NCBI Sequence Read Archive (SRA) data repository under the BioProject ID PRJNA1166155.
