# Supplementary material for: Glandless, a tomato HD‐ZIP transcription factor, is important for the gland formation of type VI trichomes
Source: Plant J. 2025 Jul 2;123(1):e70308. doi: 10.1111/tpj.70308 (PMC12223484; doi:10.1111/tpj.70308)
Supplement: Supplementary file 5 — Figure S1. Close‐up images of different types of trichomes on leaves and stems. Figure S2. Leaf trichome densities. Figure S3. Sepal trichome densities. Figure S4. Volatile mono‐ and sesquiterpenes in leaves, stems, and isolated type VI trichomes. Figure S5. Acylsugar levels on leaves. Figure S6. Rhodamine B staining of trichomes. Figure S7. Multiple sequence alignment of SlHDZ38 (Solyc09g008810) gDNA. Figure S8. Sequence alignment of SlHDZ38 (Solyc09g008810) cDNA. [file TPJ-123-0-s003.pdf]

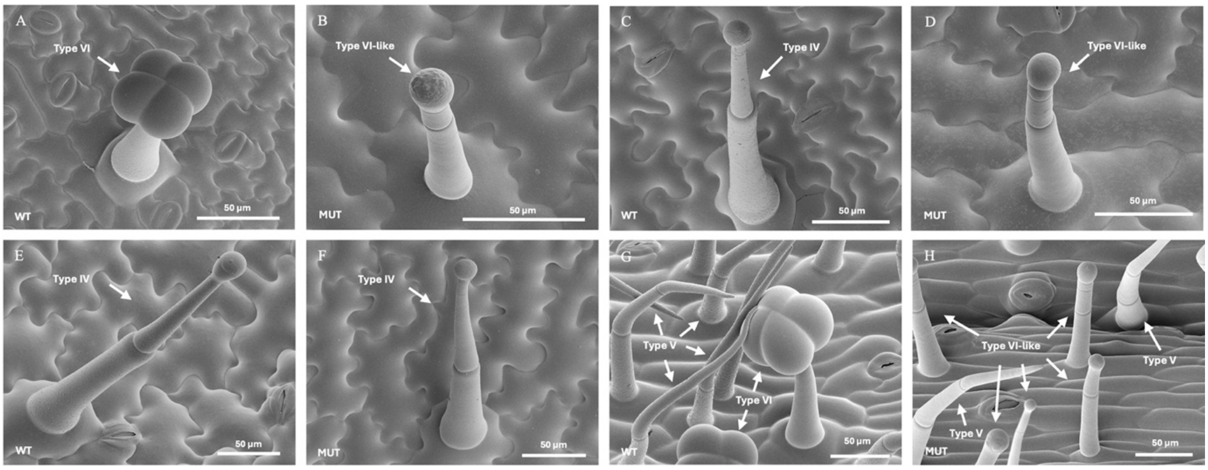

Supplemental Figure 1. Close up images of different types of trichomes on leaves and stems. *Cryo-SEM images of wild-type (WT) and glandless mutant (MUT) leaves (A, B, D), cotyledons (C, E, F) and stems (G-H). Arrows indicate different types of trichomes.*

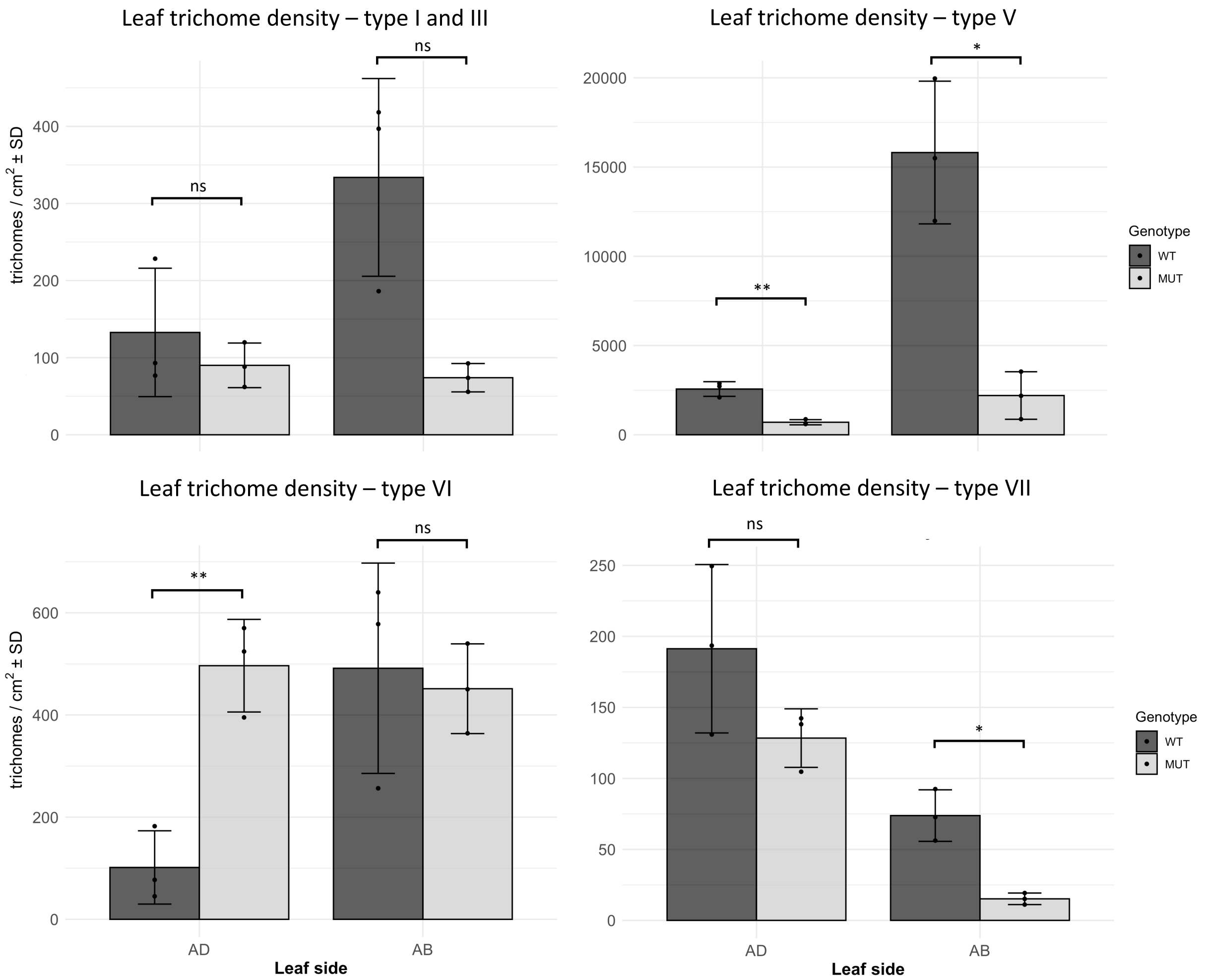

Supplemental Figure 2. Leaf trichomes densities. *Type I and III (A), type V (B), type VI (C) and type VII (D) trichomes densities on adaxial (AD) and abaxial (AB) sides of glandless mutant (MUT) and wild-type (WT) leaves. The bars represent the mean values ± standard deviation (SD) of each trichome type density, calculated from scanning-electron micrographs (0.6 cm diameter) of leaves from different plants (n=3). Differences in bars are annotated accordingly to the results of independent t-tests after Shapiro-Wilk's normality test and F-test comparison of variances (* p<0.05, ** p<0.01, ns non-significant). Significance for non-normally distributed subset was assessed with Wilcoxon non-parametric test.*

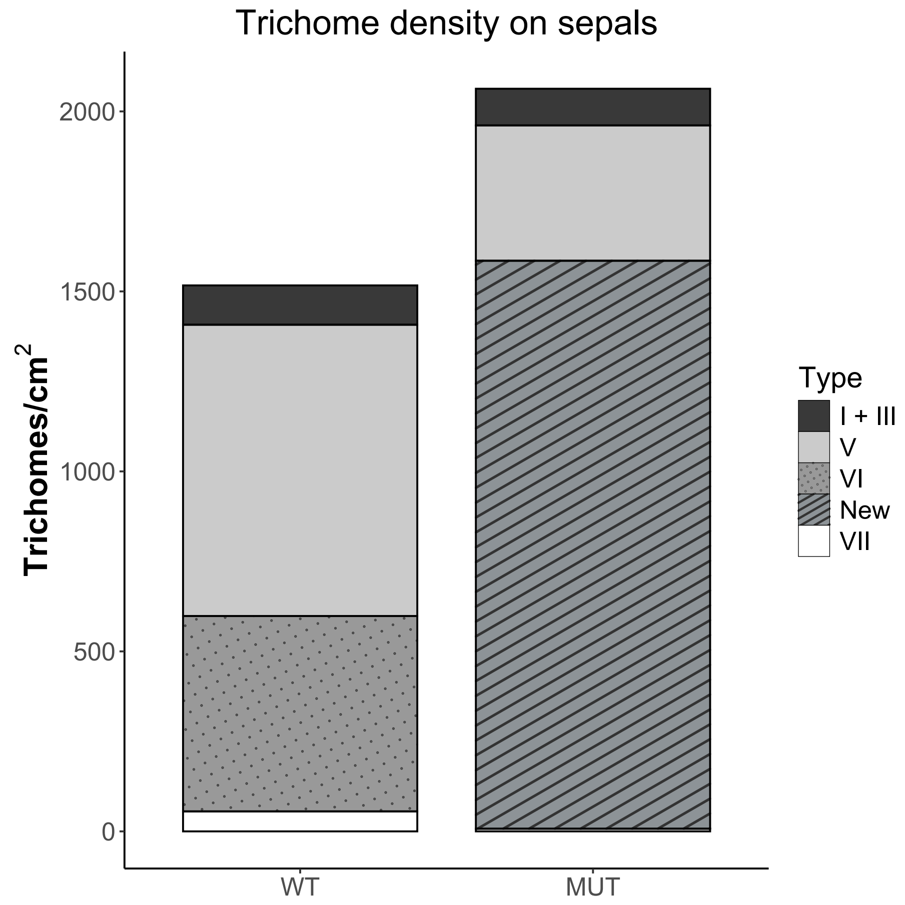

Supplemental Figure 3. Sepal trichome densities. *Densities of type I, II, III, type V, type VI and type VII on sepals of wild-type (WT) and glandless mutant (MUT) plants.*

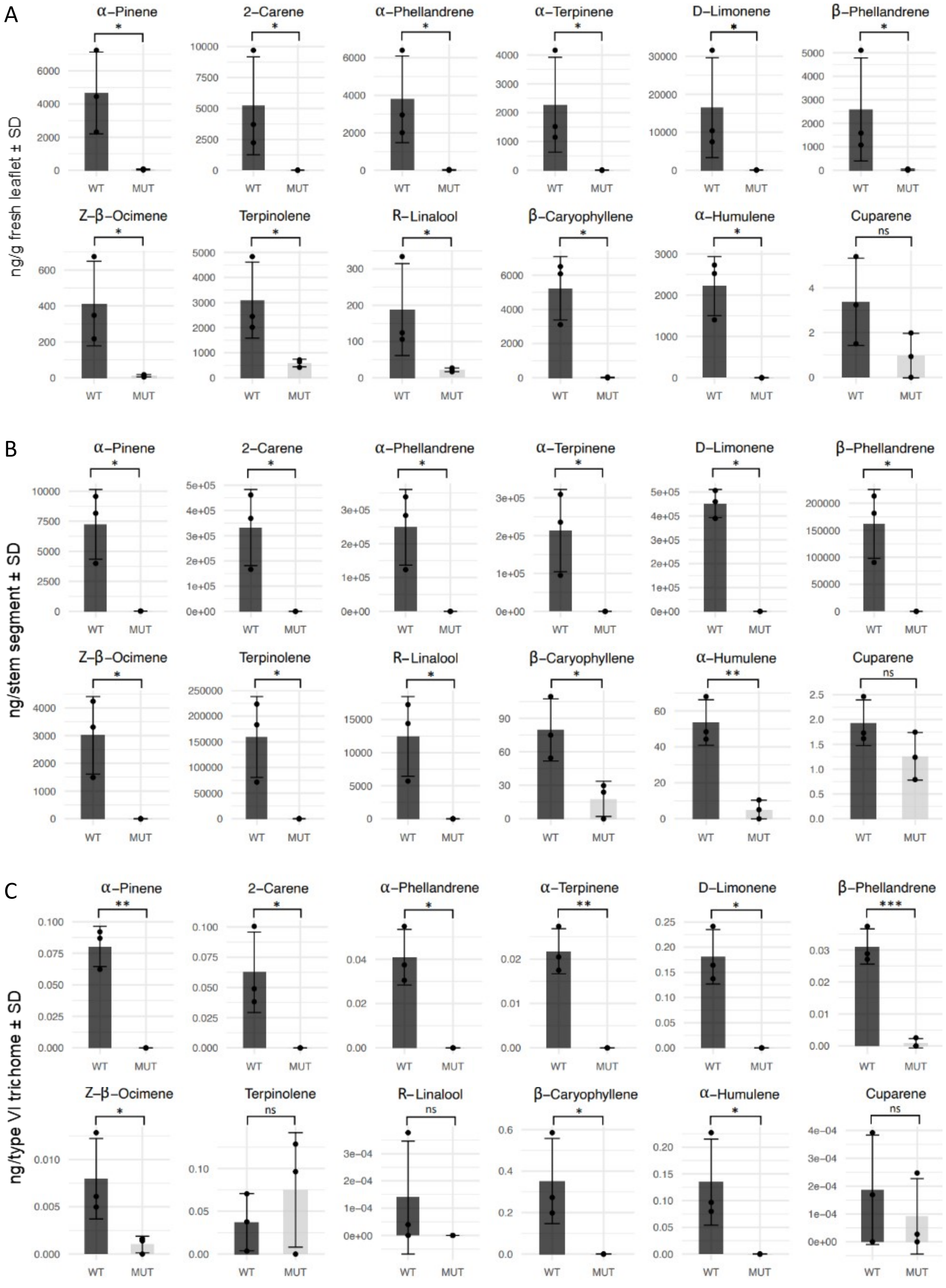

Supplemental Figure 4. Volatile mono- and sesquiterpenes in leaves, stems and isolated type VI trichomes. *Mono- and Sesquiterpene levels in leaves (A), stems (B) and isolated type VI trichomes (C) of wild-type (WT) and glandless mutant (MUT) tomato plants (n=3). The bars represent the mean values ± standard deviation (SD) of target volatile terpene level quantified by GC-MS and normalized by stem length, leaf fresh weight and number of isolated type VI trichomes. Differences in bars are annotated accordingly to the significance levels resulting from independent t-tests after Shapiro-*

Wilk's normality test and F-test comparison of variances (p<0.05, ** p<0.01, *** p<0.001 ns non-significant). Significance for non-normally distributed subset was assessed with Wilcoxon non-parametric test.*

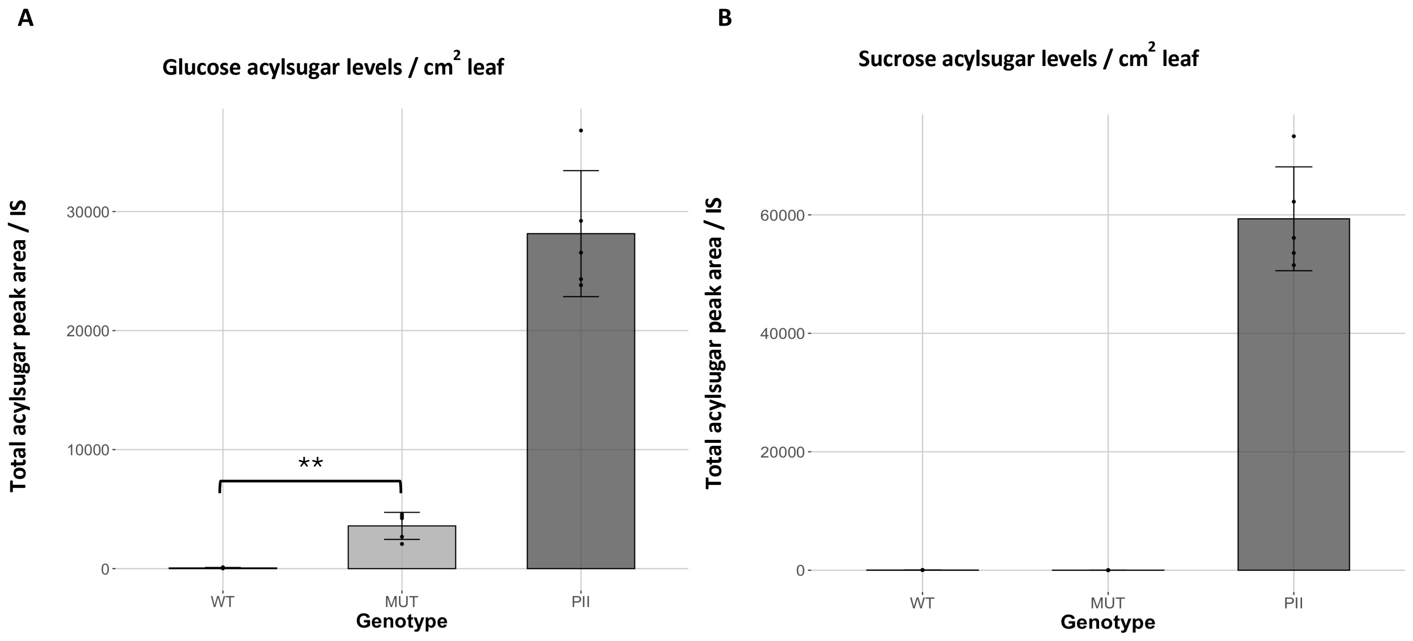

Supplemental Figure 5. Glucose and sucrose acylsugar levels in leaves and stems. *Total glucose acylsugar (A) and sucrose acylsugar (B) levels in leaves per cm^2 tissue of wild-type (WT) & glandless mutant (MUT) tomato plants, together with Solanum pennnellii (PII) accession LA716 as a positive control (n=5). The bars represent the mean values ± standard deviation (SD) of the sum of the levels of target acylsugars quantified by LC-MS. Differences in bars are annotated accordingly to the significance levels resulting from independent t-tests after Shapiro-Wilk's normality test and F-test comparison of variances (* p<0.05, ** p<0.01). Significance for non-normally distributed subset was assessed with Wilcoxon non-parametric test.*

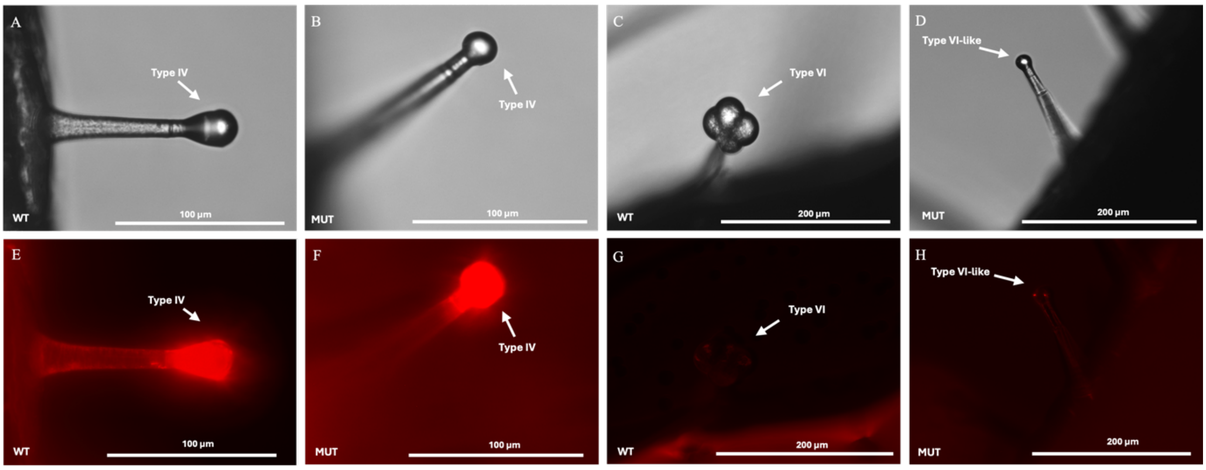

Supplemental Figure 6. Close up images of different types of trichomes on cotyledons and mature leaves. *Different types of trichomes on wild-type (WT) and glandless mutant (MUT) plants stained with Rhodamine B as seen with an EVOS inverted light microscope without filter (A-D), and using the Texas Red filter (E-H). Type IV trichomes were located on cotyledons (A-B, E-F), whereas type VI and VI-like trichomes were found on mature leaves (C-D, G-H). Arrows indicate different types of trichomes.*

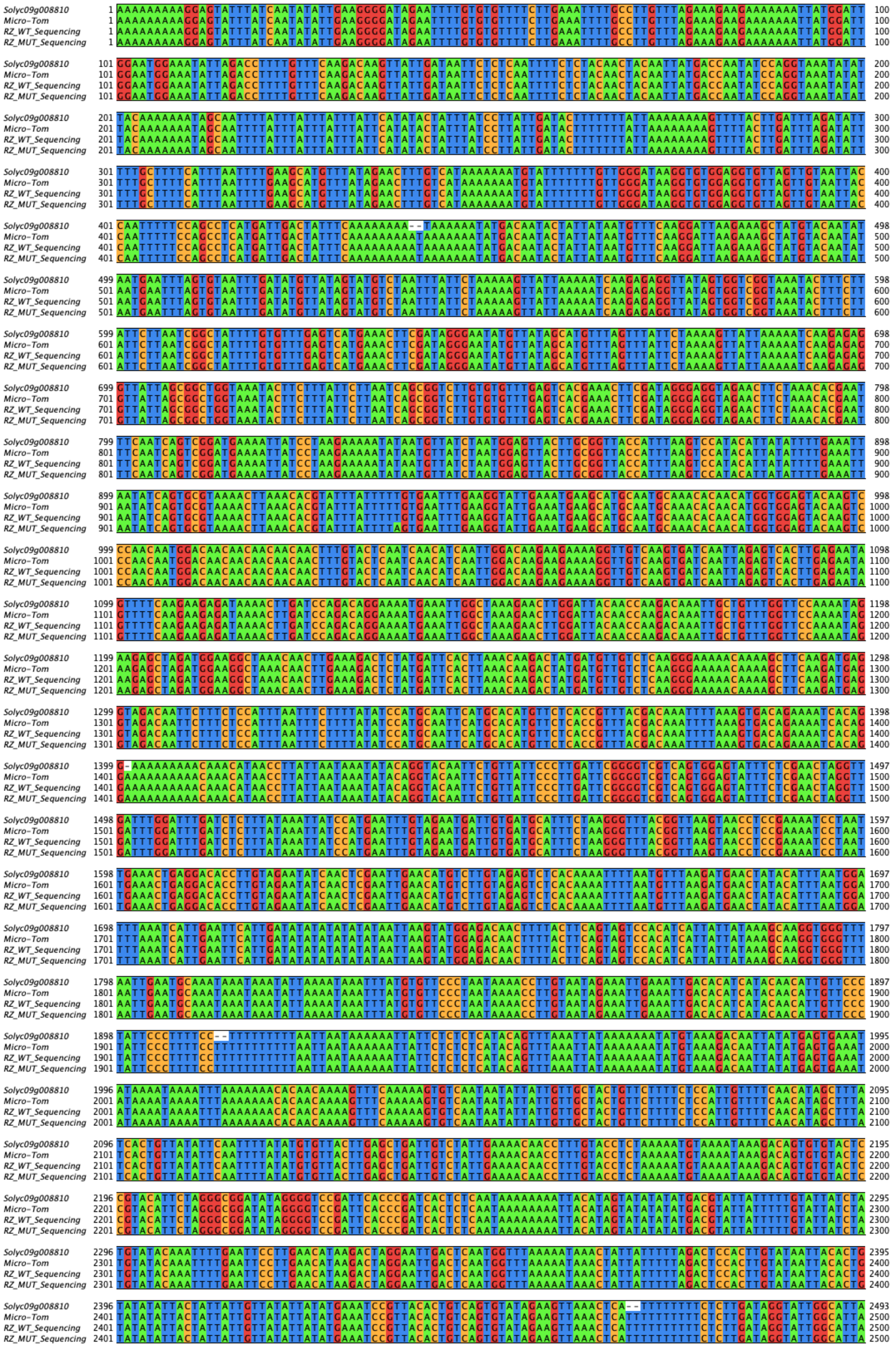
Solyc09g008810
Micro-Tom
RZ_WT_Sequencing
RZ_MUT_Sequencing

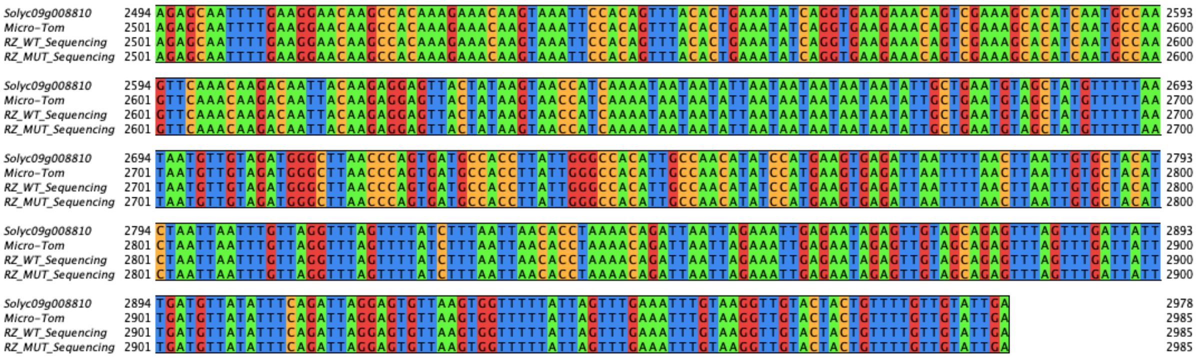

Supplemental Figure 7. Multi sequence alignment (MAFFT) of SlHDZ38 (Solyc09g008810). *MAFFT of SlHDZ38 gene sequence in the tomato genome assembly SL4.0 (https://solgenomics.net), in the Micro-Tom genome assembly (BioProject PRJNA1050426, Solanum lycopersicum cv Micro-Tom) and in the sequencing results of RZ wildtype and glandless mutant (T938A substitution).*

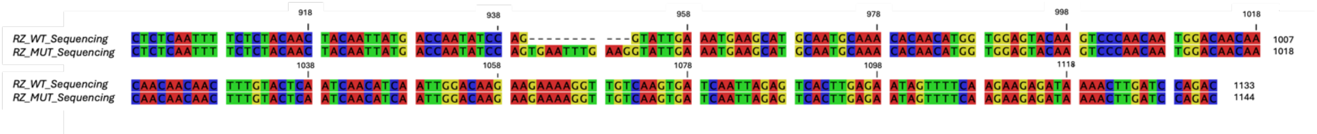

Supplemental Figure 8. Sequence Alignment of SlHDZ38 (Solyc09g008810) cDNA in RZ2 wildtype and glandless mutant around exon 2 showing a different splicing in the glandless mutant.
