# Supplementary material for: Glandless, a tomato HD‐ZIP transcription factor, is important for the gland formation of type VI trichomes
Source: Plant J. 2025 Jul 2;123(1):e70308. doi: 10.1111/tpj.70308 (PMC12223484; doi:10.1111/tpj.70308)
Supplement: Supplementary file 6 — Data S1. Supplemental methods: Mapping of the glandless mutation; acylsugar measurements. [file TPJ-123-0-s005.pdf]

Supplementary methods:

Mapping of the glandless mutation.

We indeed used a different EMS-population than in Xu et al, 2018, the RZ-2 cultivar. The backcrosses were made to the RZ-2 cultivar: a plant fixed for the *glandless* mutation was generated by inbreeding and phenotypic selection. This individual was 2 times crossed with RZ2, and then selfed. For QTL-Mapping: 90 F2 individuals were screened with 196 markers; for fine-mapping cycle 1: 96 individuals with 7 markers; for fine-mapping cycle 2a: 512 individuals were screened for recombinants with 3 markers; for fine-mapping cycle 2b: 31 recombinants fine-mapped in higher resolution with 8 markers.

The actual coordinates of fine-mapped region are SL3.0 ch09: 2.069.076 – 2.290.464 that includes 27 genetic annotations (ITAG3.0). The parents of glandless only contain 35 polymorphisms in the target locus, of which 30 are located intergenic and 5 are located in-gene. All the in-gene polymorphisms are located in introns. Polymorphisms were identified by whole genome sequencing of both parents of *glandless*, mapping to reference genome SL3.0, subsequent SNP-calling and comparison of polymorphisms between the parents of *glandless*. From the in-gene polymorphisms, 4 are located in stretches of A/T nucleotides, more than 100 nucleotides from the nearest exon. Mutations between the parents of *glandless* located intergenic or in stretches of A/T nucleotides in introns were considered less likely to impact protein functionality, and therefore less likely to induce the aberrant trichome phenotype. Another polymorphism was located just outside of the 2nd exon of SlHDZ38 (Solyc09g008810). The sequence adjacent to this mutation contains the nucleotide pattern associated with splice acceptors. This mutation is hypothesized to introduce a premature splice acceptor site. We sequenced the cDNA amplicon the glandless mutant to show the aberrrant splicing (Supplemental figure 8) .

We did confirm that the sequences of the SlHDZ38 gene are identical in the tomato genome assembly SL4.0 (https://solgenomics.net), in the Micro-Tom genome assembly (BioProject PRJNA1050426, Solanum lycopersicum cv Micro-Tom) and in the sequencing results of RZ2 wildtype. We also compared these three sequences with the sequencing result of RZ2 mutant *glandless*. The results are shown in form of a multi sequence alignment that is provided in the manuscript (Supplemental figure 7). A few nucleotide differences are found between the *SlHDZ38* gene in the tomato genome assembly SL4.0 and the other sequences, but these are located in intronic regions. No differences are therefore expected in the (predicted) protein sequences between these tomato accessions.

Analysis of acylsugar content

To analyse the acylsugar content, 6 leaf disks (3.02 cm^2) were taken from the second pair of leaflets of the fourth true leaf counting from the bottom. The leaf disks were then

placed in 750 µl acetonitrile/isopropanol/water (3:3:1, v/v/v) solution with propyl 4-hydroxybenzoate (10µg/ml) as internal standard. After 60 minutes, the supernatant was pipetted in a 1.5mL safe-lock tube (Eppendorf® Safe-Lock).

Supernatant was then filtered on Acrodisc® 0.2 µm WWPTFE filter. 300 µl of filtrate was pipetted into a 1.5 mL glass vial with 300 µl insert. Metabolites were separated by UHPLC system (Waters® Acquity H-class) using an auto-sampler set at 5°C. 0.5 µL of extract was injected onto UHPLC column (Waters Acquity® HSS C18 100 mm x 2.1 mm, 1.8 µm) maintained at 40°C. The mobile phase solution was composed of acetonitrile/water (95:5,v/v)(solution A) and water/acetonitrile (95:5,v/v)(solution B), both with 0.1% formic acid (v/v). The elution flow rate was 0.3 mL/min with the following gradient: B: 0−2.90 min, 100−50%; 2.90−16.17 min, 50−0%; 16.17−30.00 min, 0%; 30.00−35.00 min, 0−100%; and equilibration from 35.00–41.00 min, 100% B. The UHPLC system was coupled with a Time of Flight-Mass spectrometer (Waters® Xevo G-3) equipped with an electrospray ionization source in negative mode. Source temperature was set at 120°C. The ion transfer capillary temperature was set at 450°C and needle voltage at 1.0 kV. Nitrogen was used as nebulizing gas with the desolvation gas flow rate at 800L/h and cone gas flow rate at 25L/h. The spectra were acquired within mass ranging from 50−1200 Da, with no collision in low energy and ramp collision from 15 to 30 in high energy. Data acquisition and processing were performed using MassLynx™ and TargetLynx™ software. Results were normalized for propyl 4-hydroxybenzoate equivalent in peak area per cm^2 leaf area.
